# Dissemination of the Omicron Variant and Its Sub-Lineages among Residents and Travelers in Its First Year of Emergence in Venezuela

**DOI:** 10.3390/v15071460

**Published:** 2023-06-28

**Authors:** Zoila C. Moros, José Luis Zambrano, Yoneira Sulbaran, Carmen L. Loureiro, Ernestina Marulanda, Francis Bracho, Pierina D’Angelo, Lieska Rodríguez, Ferdinando Liprandi, Héctor R. Rangel, Rossana C. Jaspe, Flor H. Pujol

**Affiliations:** 1 Laboratorio de Virología Celular, Centro de Microbiología y Biología Celular, Instituto Venezolano de Investigaciones Científicas, Caracas 1020, Venezuela; zcmoros@gmail.com (Z.C.M.); jlzr.ivic@gmail.com (J.L.Z.); 2 Laboratorio de Virología Molecular, Centro de Microbiología y Biología Celular, Instituto Venezolano de Investigaciones Científicas, Caracas 1020, Venezuela; yfsulbara@gmail.com (Y.S.); cloureir@gmail.com (C.L.L.); hrangel2006@gmail.com (H.R.R.); 3 Laboratorio de Biología Molecular CasaLab 2020, Caracas 1052, Venezuela; eemarulanda@gmail.com (E.M.); francisbm@gmail.com (F.B.); 4 Dirección de Diagnóstico y Vigilancia Epidemiológica, Instituto Nacional de Higiene “Rafael Rangel”, Caracas 1053, Venezuela; pierinads@yahoo.com (P.D.); rodriguez.lieska2@gmail.com (L.R.); 5 Laboratorio de Biología de Virus, Centro de Microbiología y Biología Celular, Instituto Venezolano de Investigaciones Científicas, Caracas 1020, Venezuela; fliprand@gmail.com

**Keywords:** COVID-19, SARS-CoV-2, Omicron Variant of Concern, mutations, lineages

## Abstract

The emergence of the SARS-CoV-2 Variant of Concern (VOC), Omicron, has been characterized by an explosive number of cases in almost every part of the world. The dissemination of different sub-lineages and recombinant genomes also led to several posterior waves in many countries. The circulation of this VOC and its major sub-lineages (BA.1 to BA.5) was monitored in community cases and in international travelers returning to Venezuela by a rapid partial sequencing method. The specific sub-lineage assignment was performed by complete genome sequencing. Epidemic waves of SARS-CoV-2 cases were observed among international travelers during 2022, a situation not seen before December 2021. The succession of the Omicron VOC sub-lineages BA.1 to BA.5 occurred sequentially, except for BA.3, which was almost not detected. However, the sub-lineages generally circulated two months earlier in international travelers than in community cases. The diversity of Omicron sub-lineages found in international travelers was related to the one found in the USA, consistent with the most frequent destination of international travel from Venezuela this year. These differences are compatible with the delay observed sometimes in Latin American countries in the circulation of the different lineages of the Omicron VOC. Once the sub-lineages were introduced in the country, community transmission was responsible for generating a characteristic distribution of them, with a predominance of sub-lineages not necessarily similar to the one observed in travelers or neighboring countries.

## 1. Introduction

The COVID-19 pandemic, caused by SARS-CoV-2 infection, caused around 660 million cases and 6.7 million deaths worldwide until the end of 2022 [1]. The virus belongs to the family Coronaviridae. Because of its extensive replication, the virus has accumulated several mutations, which allow its classification in more than 2000 lineages. Among the different lineages of SARS-CoV-2, five Variants of Concern (VOCs) and several Variants of Interest (VOIs) have been recognized by the WHO. VOCs are variants for which enhanced transmission and/or immune escape abilities were confirmed during the epidemic, while in the VOIs, these characteristics were suspected, although not necessarily confirmed [2,3,4].

The last known VOC until the end of 2022 was Omicron. This VOC caused immediate concern for two reasons: its emergence in November 2021 was associated with an explosive increase in cases in South Africa, and it harbors a large number of mutations compared to the previous ones [5]. This VOC displaced all the other lineages circulating in the world, which belonged mainly to the Delta VOC. At the end of June 2022, the WHO designated the Omicron VOC as the only variant circulating in the world [6,7]. Several sub-lineages have been described for the Omicron VOC. The emerging sub-lineages progressively acquire more mutations. Some of these mutations are associated with enhanced transmissibility, and many of them confer to the new variants the ability to escape from the immune response acquired by vaccination or by previous infection, even with previous Omicron VOC [7].

The COVID-19 pandemic occurred in Venezuela, with a first epidemic peak around August 2020 and then a sustained peak from April to October 2021. The highest epidemic peak occurred in January 2022. In 2021, four VOCs and two Variants of Interest (VOI) were identified in Venezuela. The Gamma VOC (originated in Brazil) was the first to be detected and was responsible for the beginning of the sustained peak of 2021. This variant was first predominant in the states neighboring Brazil, and then became progressively introduced throughout the country [8]. Then, the VOI Lambda (first identified in Peru) and the VOC Alpha (first detected in the UK) were found. These VOCs were only detected at low frequencies. The VOI Mu (mainly introduced through the frontier with Colombia, the country where it emerged) was detected in May 2021. This VOI progressively displaced the Gamma VOC in the country. This VOI was, in turn, displaced by the Delta VOC, first detected at the end of June 2021: several sub-lineages of the Delta VOC could be detected circulating in 2021 in Venezuela. Finally, the Omicron VOC was identified at the beginning of December and rapidly displaced all the other circulating variants in the country [9].

Venezuela receives a relatively low number of international travelers (IT) compared to the neighboring countries. All IT arriving at Venezuelan airports were subjected to molecular testing for SARS-CoV-2 until December 2022; this testing allowed us to describe the introduction of the Omicron VOC in the country previously [10]. This study aims to describe the dissemination of the Omicron VOC lineages in Venezuela by monitoring the diversity of viral variants in community cases and international travelers returning to Venezuela.

## 2. Materials and Methods

This study was approved by the Human Bioethical Committee of IVIC. Of the five international commercial airports available in Venezuela in 2022, Aeropuerto Internacional Maiquetia Simon Bolivar (close to Caracas, the capital of the country) is the largest and was chosen for this study. Samples from this airport were evaluated by LAMP according to manufacturer instructions (Evotech-Mirai Genomics, Innopolis City, Verkhneuslonsky Region, Republic of Tatarstan, Russia). The SmartAmp SARS-CoV-2 Detection Kit targets and amplifies a specific sequence within the genomic region coding for Nsp15. LAMP was performed in a C1000 Touch thermal cycler (BioRad Life Science, Hercules, CA, USA). For sequence analysis, positive samples from January to December 2022 were retested by qRT-PCR according to manufacturer instructions (Sansure, Changsha, Hunan Province, China). Briefly, the Sansure kit consists of two components, the buffer mix containing primers and probes for the detection of SARS-CoV2 (ORF1ab and N gene) and an internal control (RnaseP), and a mix of enzymes (reverse transcriptase and Taq polymerase). The master mix consists of 26 µL of buffer mix plus 4 µL of enzyme mix and 20 µL of sample. The PCR program requires 30 min of reverse transcription and 45 cycles of amplification (96 °C/60 °C cycles) using the Fan and Rox channel for the ORF1ab and N genes, respectively, and the Cy5 channel for RnaseP. qRT-PCR was performed in a Bioer LineGene 9600 Real Time Thermalcycler PCR System (Bioer, Hangzhou, China) or a Biorad C1000 Touch Thermal Cycler (BioRad Life Science, Hercules, CA, USA). In addition, samples positive by qRT-PCR during the routine COVID-19 diagnosis in Venezuela from the same period were analyzed. The identity of the patients was maintained to be anonymous.

RNA from samples positive by qRT-PCR (with Ct below 30) was amplified with primers 75L and 76.8R to generate an amplicon of 614 nt, with the PCR conditions previously described [9,10]. RT-PCR was performed in a Bioer Gene Explorer GE-99G (Bioer, China). PCR-purified fragments were sent to the Macrogen Sequencing Service for capillary electrophoresis sequencing by the Sanger method (Macrogen, Seoul, Republic of Korea). This fragment allows us to analyze amino acids 345 to 533 of the Spike gene (S), which includes several mutations of the Omicron VOC and other variants, allowing the differentiation of the major sub-lineages BA.1 to BA.5. The 5′non-coding region of the SARS-CoV-2 genome (5′NCR) was also analyzed for differentiating the sub-lineages BA.4 and BA.5, with an amplicon of ≈640 nt generated with primers 1L and 3R [11]. Sequences from the S region and the 5′NCR were sent for sequencing with the 75L and 1L forward primer, respectively, and aligned with the DNAman program (DNAMan v 5.2.2, Lynon BioSoft, Vaudreuil, QC, Canada), for detecting mutations specific to each VOC and sub-lineage, by comparison with reference sequences of each VOC and major sub-lineage. The sequences from the 5´NCR were aligned with reference sequences of BA.4 and BA.5 isolates to detect a 9 nt deletion present in the BA.4 isolates.

Complete genome sequencing was performed on randomly selected samples (with Ct below 25) by Next Generation sequencing. Libraries were prepared with a DNA Prep library preparation kit using the Nextera DNA CD Indexes (Illumina, Inc., San Diego, CA, USA) as previously reported [9], or Illumina COVIDSeq Assay (96 Samples) (RUO Version, Document # 1000000126053 v05), using IDT for Illumina-PCR Indexes set 1 or 3. The libraries were pooled, quantified (Qubit DNA HS, Thermo Scientific, Waltham, MA, USA), and their quality checked (Bio-Fragment Analyzer, Qsep1-Lite, BiOptic, New Taipei City, China) before sequencing, which was performed with 10% PhiX control v3, using an iSeq 100 or MiSeq platform and a 300 cycle V2 kit with paired-end sequencing. Viral genome assembly was performed using the Dragen COVID-19 program or Genome Detective Virus tool. The variant assignment was performed using the Dragen COVID-19 program (Illumina, Inc., USA), Nextclade Web 1.14.1, or Pangolin COVID-19 Lineage Assigner. Nucleotide sequences of complete genomes were deposited into the GISAID database. Although the lineage assignment was mainly similar between the different programs, the sublineage shown in this study corresponds to the one assigned by GISAID (Supplementary Table S1).

Statistical differences were evaluated by Chi-Square tests; *p* values less than 0.05 were considered significant.

## 3. Results

**Figure 1:**
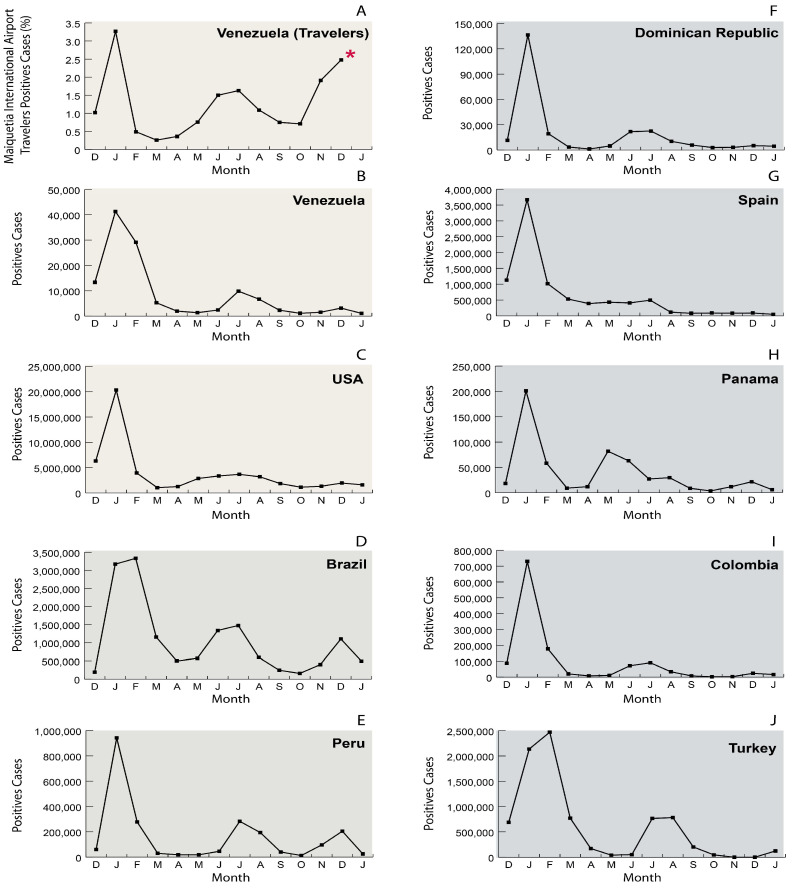
(**A**) Frequency of PRIT in international travelers returning to Venezuela. (**B**–**J**) Number of COVID-19 cases in selected countries. The Dominican Republic, Turkey, and Spain are shown since there are direct flights from Venezuela to these destinations. The graphs were drawn with RawGraph and edited with Adobe Illustrator. * End of testing of travelers returning to Venezuela.

Figure 1 shows the evolution in the number of positive SARS-CoV-2 cases in the world and selected countries and the percent positivity observed in travelers from Maiquetia airport from December 2021 to December 2022. Before December 2021, no peak of positive cases was observed among IT, with a positivity rate for international travelers (PRIT) below or near 1% [8]. In 2022, a different picture was observed in the frequency of PRIT (Figure 1A). The first Omicron wave peaked around 3.6% in January and then returned to the PRIT observed in 2021, below 1%, in March and April. An extended peak of PRIT was observed in June-August, and then PRIT peaked up to 2.5% at the end of 2022. The peaks of PRIT were not correlated with the ones observed in Venezuela (Figure 1A vs. Figure 1B): indeed, when comparing with neighboring countries or frequent destinations of IT from Venezuela, these peaks somehow correlate with the epidemic curve of the USA and Brazil (Figure 1C,D).

In order to analyze if the epidemic peaks observed in IT were associated with eventual changes in viral loads, the frequency of samples with high viral content was also analyzed during the study period. No significant difference in the number of samples with Ct inferior to 25 (high viral RNA levels) by qRT-PCR was observed during the whole year of 2022, with in average 62% of samples exhibiting Ct inferior to 25.

The frequency of Omicron VOC major sub-lineages was estimated by partial sequencing in samples from international travelers and community cases. Table 1 shows the specific mutations analyzed to identify the different Omicron VOC sub-lineages. Four common mutations were analyzed to confirm the presence of the Omicron VOC, and four others (three amino acid substitutions and one deletion) were analyzed to differentiate the five major sub-lineages of the Omicron VOC.

Complete genome sequencing confirmed the assignment of the major sub-lineages by the rapid method in most cases. The concordance between partial and complete genome sequencing methods was 95.6% for the assignment of the major sub-lineages BA.1 to BA.5 (Table 2). The most frequent discrepancies occurred in the discrimination between BA.4 and BA.5 (some BA.4 lacking the deletion in the NSP1 region) and BA.2 isolates classified as BA.5 (some BA.2 isolates harboring the mutation L452R). Nevertheless, the rapid method allowed the evaluation of a large number of samples with less than 5% error. These discrepancies were more frequently found during the emergence of new sub-lineages of the Omicron VOCs.

**Figure 2:**
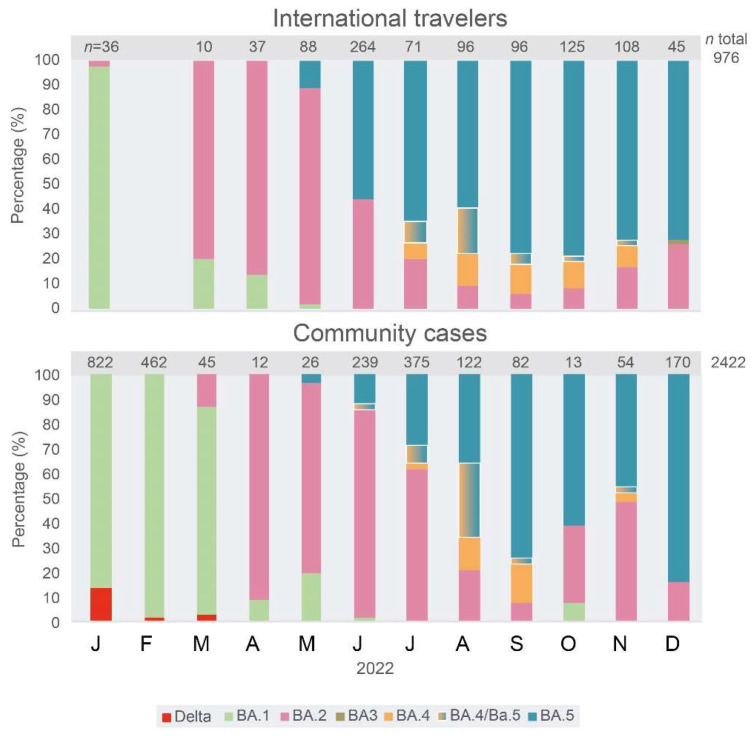
Frequency of the major sub-lineages of the Omicron VOC in international travelers and community cases from January to December 2022. Samples were classified in the major sub-lineages of the Omicron variant by partial genome sequencing, by identification of the mutations described in Table 1, and/or by complete genome sequencing. No samples were available for IT in February 2022. BA.4/BA.5: samples for which discrimination between these two sub-lineages could not be achieved, since the NSP1 region could not be amplified. The graphs were drawn with RawGraph and edited with Adobe Illustrator.

Figure 2 describes the succession of major sub-lineages of the Omicron VOC during 2022 in Venezuela, estimated by the rapid sequencing method in 3398 samples. The major sub-lineages prevailed in numerical order for the BA.1 to BA.5 sub-lineages, except for BA.3, which was almost absent. The change in the frequency of emerging sub-lineages occurred earlier, by around two months, in variants found in international travelers compared to variants in community cases (CCs). For example, the BA.2 major sub-lineage prevailed in Venezuela until July 2022, while it was displaced by the BA.5 as the major sub-lineage in IT since June. The Delta VOC was still found in some CCs until March 2022, while it was absent in IT in 2022.

**Figure 3:**
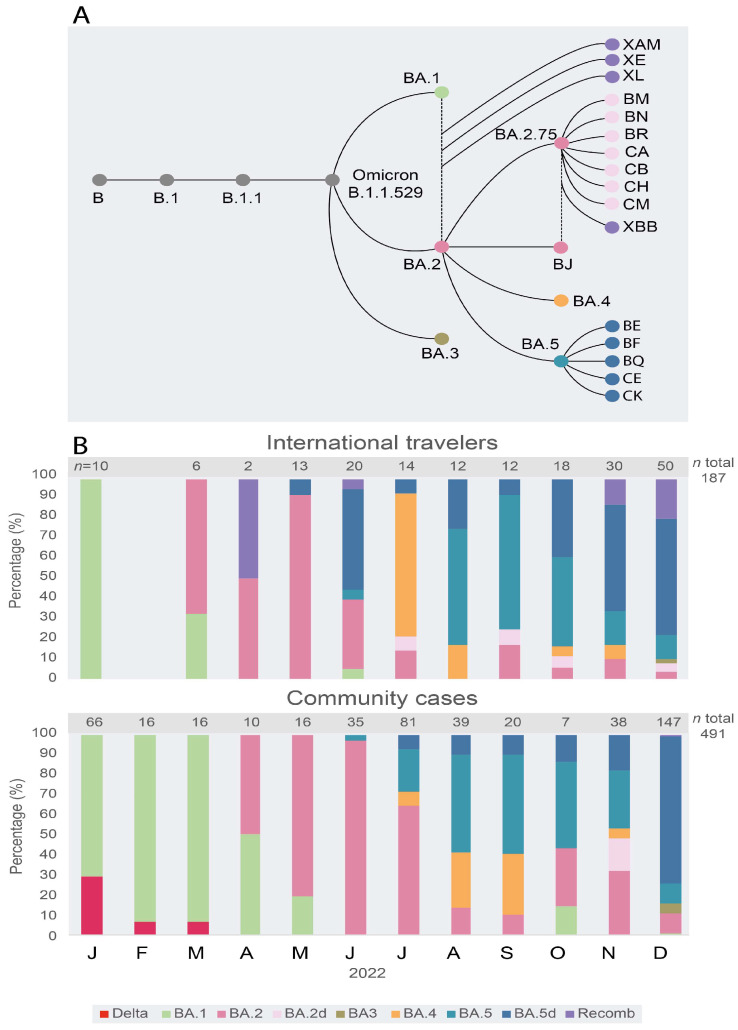
Succession of Omicron sub-lineages in international travelers and community cases from January to December 2022, as assessed by complete genome sequencing. (**A**) diagram of the evolution of sub-lineages of the Omicron VOC. Dotted lines refer to recombination events. (**B**) frequency of major sub-lineages and descendant sub-lineages (d) or recombinant (Recomb). No samples were available for IT in February 2022. The graphs were drawn with RawGraph and edited with Adobe Illustrator.

**Figure 4:**
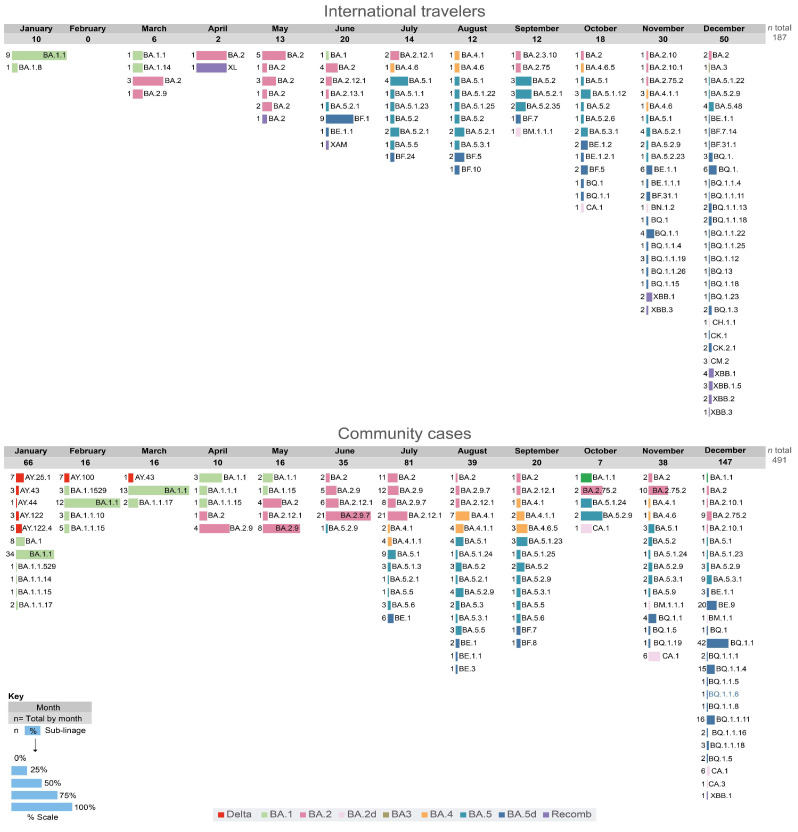
Sub-lineages of Delta and Omicron VOCs by month. The numbers below each month describe the number of samples. Samples were classified in the major sub-lineages of the Omicron variant by complete genome sequencing, and the sub-lineage was assigned according to GISAID classification. The colored bars represent the relative frequency for each sub-lineage. The graphs were drawn with RawGraph and edited with Adobe Illustrator.

A total of 678 complete genome sequences were analyzed, 187 from IT and 491 from CCs (Figure 3, GISAID accession numbers with date and locality in Supplementary Table S1). Since July 2022, a great diversity of sub-lineages has been observed by complete genome sequencing of viral samples (Figure 4). In December, a large number of complete genome sequences could be obtained. Up to 29 different sub-lineages were found in 50 IT samples, a diversity significantly higher (*p* < 0.001) than the one observed in CCs (26 different sub-lineages in 145 samples, Figure 4), a situation expected because of community transmission in CCs. 

The wider diversity of sub-lineages and recombinant forms of the Omicron VOC was always first detected in samples from IT and then in CCs in Venezuela. However, the distribution of specific sub-lineages was different in CCs compared with that observed in IT. In CCs, for example, the sub-lineage BA.2.75.2 has been frequent since October 2022, and BQ.1.1 was frequent in November and December (Figure 4), while in IT, this former lineage was never frequent (Figure 4). In the case of BQ.1 and sub-lineages, in December, the frequency of these sub-lineages was similar in CCs (59%) and IT (46%, *p* > 0.05). The BA.1.1 sub-lineage was still circulating in CCs in December 2022, but it was not found after June in IT. Only one BA.3 sub-lineage isolate was identified, in IT, relatively late in December 2022 (Figure 3B). The sub-lineages of BA.5 were the most frequent lineages in IT since June 2022, while they prevailed in CCs two months later, in August (Figure 3B). 

## 4. Discussion

Venezuela performed molecular testing of SARS-CoV-2 in international passengers arriving at the commercial airports of the country very soon after the declaration of the pandemic of COVID-19. An explosive increase in PRTI was observed since the second half of December 2021 in most of these airports, associated with the circulation of the Omicron VOC worldwide [10]. 

The PRIT curve was correlated with the number of cases from the USA and Brazil. This relation is in agreement with one of the most common destinations of Venezuelan international travel by airplane, i.e., Florida and the USA [13]. The PRIT and the RNA levels in the positive samples may be influenced by the changing regulations related to vaccination. On 1 July 2021, for example, the EU Digital COVID Certificate Regulation entered into the application [14]. The absence of COVID-19 testing requirements in vaccinated individuals may have influenced the increase in PRIT in 2022. We were also expecting that the frequency of samples with high viral RNA levels could also increase with the reduction in the testing requirements. Contrary to our expectations, the frequency of samples with high viral RNA levels remained similarly high during the entire evaluation period. 

The strategy adopted in this study (sequencing of a small genomic fragment of the Receptor Binding Domain of SARS-CoV-2 S protein, complemented with partial sequencing of the ORF1 region to differentiate BA.4 and BA.5) allowed us to analyze many samples with a good correlation (more than 95% in more than 340 complete genomes) for major sub-lineage assignment. Rapid sequencing was more economical in our hands and allowed us to analyze a higher number of samples in a shorter time. However, since July 2022, a great diversity of sub-lineages and recombinant forms has been detected, reducing the utility of the rapid sequencing method for sub-lineage monitoring. This worldwide phenomenon has been called a swarm of variants or variant soup [15], defined by the co-circulation of multiple sub-lineages of the Omicron VOC, with the appearance of common mutations, particularly associated with immune evasion, through convergent evolution [16].

The results show the influence of air travelers on the introduction of the different sub-lineages of Omicron to the country. This situation contrasts with that observed for Gamma and Mu, for which, although the introduction by air route is not ruled out, the land route seems to have played a greater role, since the first states with the highest frequency of these variants were the border states, the Eastern border for Gamma VOC and Western one for Mu VOC, according to the countries of origin of these variants, Brazil and Colombia, respectively [9,16]. However, the introduction by land of the Omicron sub-lineages, although not evaluated in this study, certainly occurred and possibly influenced the distribution of the viral variants in the country. A limitation of this study is the number of positive samples that could be tested in 2022 compared to 2021. This is due to the fact that after the unprecedented spike in cases due to the first wave of the Omicron variant from December 2021 to January 2022, a significant reduction in cases occurred in the country. Afterward, despite a probable consistent number of cases restarting in July 2022, fewer people sought a diagnosis. This might be due in part to the combination of the eventual reduction in morbidity associated with the Omicron variant, compared to the Delta variant, for example, and with the important herd immunity that was probably prevalent throughout the country [7,17,18,19,20]. This implied that the waves of the major sub-lineages could not be evaluated by geographic regions in the country, so the study was limited to evaluating the country as a single region.

Even if the number of complete genomes is relatively reduced, we could appreciate a high diversity of sub-lineages circulating in Venezuela, with one or two major clades each month in CCs. Two sub-lineages were found frequently since the second half of the year 2022: BA.2.75.2 and BQ.1.1 and descendants of BQ.1; this latter, in turn, was derived from the BA.5 major sub-lineage [16,21]. BA.2.75.2 and BQ.1.1 harbor several mutations associated with reduced neutralization both by sera from immunized individuals and by several monoclonal antibodies [22]. They were reported among the ones more resistant to the bivalent BNT162b2 vaccine-induced neutralizing antibodies [23]. The circulation of these sub-lineages agrees with a probable high level of herd hybrid immunity in the Venezuelan population in the second semester of the year 2022, after two waves of the Omicron variants in the country (Figure 1).

The analysis of the frequency of sub-lineages in the main neighboring countries is not similar to the frequency of sub-lineages found in Venezuela. In Colombia, the sub-lineage BA.2.75.2 did not surpass 8% prevalence during 2022, although the sub-lineage BQ.1 did predominate (up to 85% in the middle of December 2022) [24]. A pattern similar to the one observed in Colombia was observed in Brazil, where the frequency of BA.2.75.2 did not surpass 2% and up to 78% for BQ.1.1 [24].

Another important sub-lineage, XBB.1.5, emerged in the USA at the end of 2022 and predominated in February–March 2023. The XBB.1.5 sub-lineage is a recombinant form of two BA.2 sub-lineages. It exhibits high immune evasion as other previous sub-lineages but was also considered in March 2023 as the most transmissible of all the sub-lineages known to this date, surpassing the BQ.1.1 [25]. It began to be found in South America in January 2023 and predominated in the region in March 2023. This sub-lineage was not found in community cases in Venezuela until February 2023 [26], although it was found in a few international travelers since December 2022. This sub-lineage largely predominated in May 2023 in the world [24]. However, molecular and phylodynamic studies do not predict a particularly high risk of XBB.1.5 expansion to become a new global public health threat, although the acquisition of additional mutations cannot be ruled out [27]. 

Then, once the “soup of sub-lineages” was introduced in the country, community transmission was responsible for generating a characteristic distribution of them, with greater frequency of certain sub-lineages, not necessarily similar to those that predominated in travelers or neighboring countries.

## 5. Conclusions

International travelers returning to Venezuela were probably a significant source of the introduction of the Omicron sub-lineages in the country. A great diversity of sub-lineages was found both in IT and CCs, particularly after July 2022. However, once the different lineages were introduced in the country, some of them were disseminated preferentially in the country at the end of 2022, such as BA.2.75.2 and BQ.1.1.

## Figures and Tables

**Table 1:** Mutations analyzed by Sanger sequencing for assignment of Omicron major sub-lineages.

Variant	BA.1	BA.2	BA.3	BA.4	BA.5
S Common *					
K417	N	N	N	N	N
T478	K	K	K	K	K
E484	A	A	A	A	A
N501	Y	Y	Y	Y	Y
S Specific *					
D405		N	N	N	N
R408		S		S	S
L452				R	R
ORF1					
aa 141–143				Del **	

* Mutations in the S protein used for discrimination of Omicron major sub-lineages are shown. S common: 4 mutations, K417N, T478K, E484A, and N501Y, common to all sub-lineages of Omicron VOC (except in some BA.3 isolates), were chosen for confirmation of variant assignment. S specific: 3 mutations were analyzed to discriminate between the 5 Omicron major sub-lineages. ** A deletion of 3 amino acids in the ORF1 gene, present in BA.4, allows discrimination between BA.4 and BA.5. Mutation information is available in [12].

**Table 2:** Concordance between rapid and complete genome sequencing for major sub-lineage assignment.

Major Sub-Lineage	BA.1	BA.2	BA.4	BA.5	Total
NGS sequences (n)	96	105	18	124	343
Partial sequences (n)	96	96	16	120	328
Concordance	100%	91%	89%	97%	95.6%

## Data Availability

The complete genome sequences have been deposited in the GISAID database.

## Supplementary Materials

The following supporting information can be downloaded at: https://www.mdpi.com/article/10.3390/v15071460/s1, Table S1: GISAD accession number for all the complete genome generated in this study.
